# Human Brain-Based Models Provide a Powerful Tool for the Advancement of Parkinson’s Disease Research and Therapeutic Development

**DOI:** 10.3389/fnins.2022.851058

**Published:** 2022-05-16

**Authors:** Sarah F. McComish, Adina N. MacMahon Copas, Maeve A. Caldwell

**Affiliations:** ^1^Department of Physiology, Trinity Biomedical Sciences Institute, Trinity College Dublin, Dublin, Ireland; ^2^School of Medicine, Trinity Biomedical Sciences Institute, Trinity College Dublin, Dublin, Ireland; ^3^Trinity College Institute of Neuroscience, Trinity College Dublin, Dublin, Ireland

**Keywords:** Parkinson’s disease, midbrain organoids, pluripotent stem cells, human based models, transplant therapies

## Abstract

Parkinson’s disease (PD) is the second most common neurodegenerative disease and affects approximately 2–3% of the population over the age of 65. PD is characterised by the loss of dopaminergic neurons from the substantia nigra, leading to debilitating motor symptoms including bradykinesia, tremor, rigidity, and postural instability. PD also results in a host of non-motor symptoms such as cognitive decline, sleep disturbances and depression. Although existing therapies can successfully manage some motor symptoms for several years, there is still no means to halt progression of this severely debilitating disorder. Animal models used to replicate aspects of PD have contributed greatly to our current understanding but do not fully replicate pathological mechanisms as they occur in patients. Because of this, there is now great interest in the use of human brain-based models to help further our understanding of disease processes. Human brain-based models include those derived from embryonic stem cells, patient-derived induced neurons, induced pluripotent stem cells and brain organoids, as well as post-mortem tissue. These models facilitate *in vitro* analysis of disease mechanisms and it is hoped they will help bridge the existing gap between bench and bedside. This review will discuss the various human brain-based models utilised in PD research today and highlight some of the key breakthroughs they have facilitated. Furthermore, the potential caveats associated with the use of human brain-based models will be detailed.

## Introduction

Parkinson’s disease (PD) is the second most common neurodegenerative disease. It was first described in 1817 by James Parkinson in “An essay on the shaking palsy” (Parkinson, 2002). PD is currently thought to affect almost one million people in the United States alone, with incidence estimated to rise to 1.2 million people by 2030 (Marras et al., 2018). A community-based study carried out in China calculated the prevalence of PD as 1.37% in the over 60s population (Qi et al., 2021), however incidence is thought to be as high as 2-3% of the population over the age of 65 (Crompton et al., 2021). PD is attributed to the progressive loss of dopaminergic (DA) neurons from the *substantia nigra pars compacta* (SNpc) of the midbrain which has been hypothesized to be due to the toxic effects of abnormal α-synuclein (α-syn) protein aggregations known as Lewy bodies (Wakabayashi et al., 2000). α-syn associated pathology has been shown to result in altered mitochondrial metabolism, and proteasomal and autophagy-lysosomal dysregulation, ultimately leading to the death of DA neurons in the SNpc (Michel et al., 2016) [for review see Roberts and Brown (2015) and Zhang et al. (2018)]. The underlying cause of this devastating cellular breakdown has been the subject of much research over the past decades. The characteristics of DA neurons which have been proposed to result in the vulnerability of this cell population include: elaborate neuronal arborisation, numerous vesicular release sites which are enriched in α-syn, high levels of intracellular calcium due to the autonomous pacemaker activity of DA neurons and high basal levels of mitochondrial oxidative stress (Chan et al., 2010; Pacelli et al., 2015).

Clinically, PD patients present with motor symptoms such as tremor, rigidity, postural instability, bradykinesia, loss of co-ordination and shuffling or freezing gait. Non-motor symptoms associated with PD include depression, anxiety, constipation, sleep disturbances, hyposmia, paraesthesia, and cognitive and behavioural abnormalities (Jankovic, 2008; Schapira et al., 2017; Santos-García et al., 2021). Clinical diagnosis is based on the presence of bradykinesia along with resting tremor or rigidity for initial parkinsonism diagnosis. Further analysis of absolute exclusion, “red flags,” and absolute inclusion criteria are carried out before an established PD or probable PD diagnosis can be given (Postuma et al., 2015). Owing to the so-called prodromal phase occurring 20 or more years prior to symptom onset, PD is typically diagnosed upon onset of motor symptoms, at which point up to 70% of the DA neurons are lost due to disease manifestation (Goldman and Postuma, 2014).

In 1996, a number of mutations in the *SNCA* gene, which encodes the α-syn protein, were identified as risk factors for PD (Polymeropoulos et al., 1996; Nussbaum and Polymeropoulos, 1997). Since then numerous other pathogenic mutations have been identified as risk factors for PD, namely variants in the *PARKIN*, *PINK1*, *DJ-1*, *LRRK2* and *GBA* genes [for review see MacMahon Copas et al. (2021)]. However, following much research into the underlying mechanisms of PD huge gaps in our understanding of this disease still remain.

The current therapies applied to manage the symptoms of PD include Levodopa (a dopamine precursor aimed at increasing dopamine levels in the brain), monoamine oxidase B (MAO-B) inhibitors and dopamine receptor agonists (Goetz and Pal, 2014). Deep brain stimulation has had some success at managing tremor in PD patients [reviewed by Dayal et al. (2017)]. However, these approaches simply target the symptoms of PD without altering disease progression. There are currently no disease-modifying therapies available for PD. The lack of potential therapies proceeding to Phase II clinical trials and beyond might be attributed to a lack of representative human models to complement pre-clinical research using animal models. Human microglia for example, express numerous disease genes which lack suitable orthologues or are not expressed at all in other mammals (Smith and Dragunow, 2014). While these genes might be forcibly introduced and expressed in transgenic rodent models, they cannot fully recapitulate the disease as it presents in humans (Burns et al., 2015; Geirsdottir et al., 2019; Fattorelli et al., 2021). Perhaps furthering our understanding of PD is limited by the currently available animal models, and human brain-based models should instead be utilised to their full potential. This review will discuss the human models currently available and utilised in PD research including patient post-mortem tissue, human pluripotent stem cells and midbrain organoids. The strengths of these models and the discoveries they have led to will be highlighted, and the limitations associated with these models that need to be addressed for future improvements will also be discussed.

## Post-Mortem Tissue

Human post-mortem tissue has been studied in the field of PD since the late 1950’s, starting with two landmark publications. Carlsson first suggested that dopamine might be a transmitter in the brain and that it could be involved in motor function (Carlsson, 1959), and Elhringer and Hornykiewicz demonstrated a reduction in the level of dopamine in the striatum of patients suffering from idiopathic PD [for review see Lees et al. (2015)]. Interestingly 13 years later, in 1973, it was shown that there was a correlation between the concentration of dopamine in the striatum and loss of dopamine neurons in the SNpc (Bernheimer et al., 1973). This study demonstrated that the classical symptoms of PD manifest themselves when 80% of the normal dopamine concentration was lost in the striatum but this correlated to 50% loss of dopamine neurons in the SNpc, suggesting that perhaps a subset of these neurons remain intact but may be functionally impaired (Bernheimer et al., 1973). Indeed, this particular subset might have the potential to be a good target for restorative therapies [for review see Hartmann (2004)]. In addition, post-mortem tissue was utilised to determine that Lewy bodies were primarily composed of the protein α-syn (Spillantini et al., 1997) and that α-syn triplication results in the doubling of α-syn protein expression (Singleton et al., 2003; Farrer et al., 2004). Furthermore, using correlative high-resolution imaging and biophysical approaches, finer details of Lewy body structure was detailed as a crowded environment of membranes including vesicular structures and dysmorphic organelles (Shahmoradian et al., 2019).

Post-mortem tissue has been very useful in determining the value of experimental therapeutic approaches for PD such as grafting of foetal DA neurons. This includes information on their survival post transplantation, the innervation of the graft and has also provided evidence of Lewy body pathology within grafts of some recipients that received transplants at least 10 years previously [for review see Li and Li (2021)]. In a different human study, post-mortem tissue from two patients that had been given AAV2-neuturin gene therapy 8 and 10 years previously demonstrated persistent transgene expression following delivery to the CNS albeit limited with between 3 and 12% coverage of the putamen (Chu et al., 2020). Taken together these studies demonstrate the value of post-mortem tissue to inform experimental therapies and so provide a reference point for future trials.

However, there are many limitations to the use of post-mortem tissue in research which include end stage disease, post-mortem delay, aged tissue and quite often patients will have been on chronic treatments over a protracted period of time. Bearing these limitations in mind use of post-mortem tissue cannot predict processes which might be present during earlier stages of the disease, so one has to consider if any changes observed at this late stage are a cause or a consequence of the disease pathology. In order to try and circumvent such limitations, there are many animal models available to study PD, however it is worth noting that none of them faithfully recapitulate the disease process, and the anatomical organisation of the nigrostriatal pathway differs significantly from lower order species (Hardman et al., 2002). Therefore, post-mortem tissue remains necessary for verifying the usefulness of experimental therapies and for verifying results obtained from human and rodent *in vitro* and from animal models of PD.

Another potential source of human primary tissue can be obtained during a surgical procedure. However, once removed from their *in vivo* environment these cells rapidly undergo transcriptomic and phenotypical changes (Gosselin et al., 2017). There are also limitations to the use of resected tissue as it always comes from a patient with no relevant healthy control. The patient in question typically has a tumour or epilepsy and not a neurological disorder such as PD and also the available tissue is usually limited to the temporal cortex and hippocampus (Kramvis et al., 2018). This limits the usefulness of this tissue for studying neurological disorders.

## Human Midbrain Culture Models of Parkinson’s Disease

### Induced Neuron Models in Parkinson’s Disease

The direct conversion of somatic cells (e.g., dermal fibroblasts) to neurons represents a potential source of these cells for disease modelling. This technique of induced neurons was first described in 2010 by Vierbuchen et al. (2010). This was closely followed in 2011 by Pfisterer et al. (2011) and later by Caiazzo et al. (2015) who demonstrated that fibroblasts could be directly induced into dopamine neurons (iDAN) opening up the possibility of their usefulness in PD modelling. This technique has the potential advantage over stem cell-based approaches in that no conversion to a stem cell state is required in the reprogramming process, so in theory reducing the risk of tumour formation (Vierbuchen et al., 2010; Stoker and Barker, 2018). However, this process is limited by the low reprogramming efficiency of somatic tissue and hence the yield of pure iDAN produced remains low which hinders their current use in research (Han et al., 2021). Nonetheless, with an increase in our understanding of the molecular pathways involved in the differentiation of specific lineages (Colasante et al., 2019) and quantitative modelling (Merlevede et al., 2021), more efficient methods will be developed to generate more defined populations of iDAN which will allow an expansion of their use in disease modelling and drug discovery for PD.

### Human Embryonic Stem Cells in Parkinson’s Disease

The pluripotent nature of embryonic stem cells (ESC) lend them the ability to differentiate into any cell type in the body, a feat which makes them an attractive cell source for disease modelling. Thomson et al. (1998) detailed the first method to isolate ESC from the human blastocyst. These cells were shown to express SSEA-3, SSEA-4, TRA-1-60, TRA-1-81, and alkaline phosphatase, now commonly used markers of pluripotency. This truly ground-breaking discovery allowed for the generation of human brain-based models of disease. Prior to this the only way to access human brain cells was via post-mortem brain tissues, at end stage disease. Being able to study the interaction of human cells is important for understanding the disease mechanisms underlying such pathologies.

ESC can be differentiated into terminal cell populations following embryonic developmental cues. Conveniently, neuroectoderm is often considered the “default” as it will develop in cultures without serum or primitive streak initiators. Perrier et al. (2004) first directed differentiation of DA neurons from human ESC. They achieved this by culturing cells in the presence of Sonic Hedgehog (SHH) and fibroblast growth factor 8 (FGF8), with further differentiation in the presence of glial cell-derived neurotrophic factor (GDNF), dibutyryl cyclic-AMP, and transforming growth factor (TGF)-β3. The expression of commonly used neuronal markers such as; PAX6, nestin, NCAM, SOX1, and NANOG were used to confirm cell fate. Since then, there have been numerous alterations made to protocols for the differentiation of midbrain DA neurons in order to achieve the most accurate counterpart to those A9 DA neurons of the human SNpc (Yan et al., 2005; Kriks et al., 2011; Kirkeby et al., 2012; Kim et al., 2021). Once the correct DA neuronal lineage is achieved astrocytes can be generated using astrocytic cues (Emdad et al., 2012; Li et al., 2018) as can oligodendrocytes (Nistor et al., 2005; Douvaras et al., 2014; Douvaras and Fossati, 2015). Protocols also exist for the differentiation of microglia-like cells (Muffat et al., 2016; Abud et al., 2017; Douvaras et al., 2017). Human ESC can also be altered to express mutations associated with PD pathogenesis in order to investigate the effect of these mutations on cell functionality. Isogenic lines can also be produced in order to overcome the effect of the genetic differences between cell lines on results (Wulansari et al., 2021).

Human ESC have been crucial for the development of their closely related counterparts, induced pluripotent stem cells. They have also paved the way for human cell replacement therapies and drug discovery. They have been important in researching human development and thus increasing the accuracy of protocols for the generation of PD *in vitro* models. In fact, some important preclinical work using human ESC has recently paved the way for the first human clinical trials in PD patients (Wang et al., 2018; Piao et al., 2021).

#### Potential Limitations of Embryonic Stem Cells in Parkinson’s Disease Modelling

While human ESC have facilitated major advancements in the field of PD research, and continue to play a vital role in the development of therapeutic targets and the understanding of this disease, the use of these cells present considerable challenges. A very well-known limitation of these cells is their origin and the ethical considerations that this carries with it. Isolation of human ESC, which are found within the inner cell mass of the developing embryo, results in destruction of the embryo (Lo and Parham, 2009). Despite the great contribution of these cells to research this limitation will always remain. This has made it difficult to gain access to such cells. The fact that these cells are embryonic poses the issue of their lack of epigenetic and age-related signatures obtained throughout life which may be contributing factors to disease pathogenesis and therefore the applicability of ESC for investigating such avenues of age-related diseases (Horvath, 2013; Kerepesi et al., 2021).

A further limitation of ESC is the fact that it is not possible to produce patient- and disease-specific cell lines. As a result, their use in transplantation trials requires an immunosuppression regime to prevent graft versus host disease. To generate an even more physiologically accurate model, generating cells from a PD patient would be beneficial. This is where induced pluripotent stem cells (iPSC) enter the scene and it is with thanks to all of the work carried out on human ESC that these cells were able to come about.

### Induced Pluripotent Stem Cells in Parkinson’s Disease

iPSC are adult somatic cells which assume a pluripotent state when embryonic genes—*Oct3/4*, *Sox2*, *Klf4*, and *c-Myc*—are forcibly expressed in a process called reprogramming. iPSC behave much like ESC in that they are pluripotent and capable of self-renewal. iPSC were first produced from mouse skin cells in 2006, and later from human dermal fibroblasts in 2007 (Takahashi and Yamanaka, 2006; Takahashi et al., 2007). This pioneering research was a major breakthrough in the fields of regenerative medicine, disease modelling and drug discovery, and resulted in the lead researcher Shinya Yamanaka receiving the Nobel Prize in Medicine and Physiology in 2012. iPSC may be derived from skin dermal fibroblasts, peripheral blood mononuclear cells (PBMC) and numerous other somatic cell types (Cobb et al., 2018; Vlahos et al., 2019; Castro-Viñuelas et al., 2020). This facilitates the generation of patient- and disease-specific iPSC lines for *in vitro* modelling of disease phenotypes.

The first PD patient-specific iPSC line was established by Park et al. (2008). Since this seminal publication various studies have outlined the generation of PD patient-specific lines, including those generated from patients carrying mutations in PD-associated genes such as; *SNCA*, *LRRK2*, *GBA*, and *PINK1* (Marotta et al., 2020). The ability to generate isogenic control lines has allowed researchers to account for background genetic variations that arise with the use of different parent lines and which may influence the cell phenotype (Marotta et al., 2020). The ability to use these cells for the investigation of contributions of genetic mutations to disease pathogenesis is invaluable, these cells have shone a light on dysregulated processes and pathways and their impact on cellular functioning and subsequent contribution to the underlying pathogenesis of PD.

Directed differentiation of PD patient-derived iPSC toward a midbrain fate allows for the examination of cell populations in isolation. These populations–midbrain DA neurons, astrocytes, and microglia–consist of a high level of complexity and heterogeneity and cellular variability, thereby are highly representative of heterogeneous *in vivo* populations. However, this makes the reproducibility of data with these cells difficult as they are observed to respond differently to stimuli and thus the advent of single cell transcriptomics allows for in-depth analysis of heterogeneous populations (Fernandes et al., 2020). Take for example research by Oosterveen and colleagues demonstrating the importance of SOX6 positivity in neuronal DA neuron cultures when specifying a substantia nigra (SN) cell lineage. SOX6 positive neurons demonstrate a sensitivity to mitochondrial toxins as observed in PD models, whereas their closely related OTX2 positive neurons, were observed to have a resistance to these toxins (Oosterveen et al., 2021).

Similar to human ESC, iPSC can be differentiated into ventral midbrain DA neurons (Table 1), astrocytes and even microglia (Serio et al., 2013; Muffat et al., 2016; Abud et al., 2017; Douvaras et al., 2017; Haenseler et al., 2017; Nolbrant et al., 2017; Pandya et al., 2017; Takata et al., 2017; Crompton et al., 2021; Oosterveen et al., 2021). iPSC have been utilised in the investigation of disease not only at the single cell level but also through co-culture, tri-culture and even microfluidic devices. Di Domenico and colleagues were able to observe that LRRK2 mutation carrying PD patient iPSC-derived astrocytes contributed to a disease phenotype in control iPSC-derived DA neurons. However, when PD DA neurons were co-cultured with control iPSC-derived astrocytes this disease-associated phenotype was partially ameliorated (di Domenico et al., 2019). This highlights the importance of these cellular interactions in the underlying pathogenesis of disease and how crucial it is to have accurate models to fully appreciate the complexities of this disease. Furthermore, Iannielli et al. (2019) established a co-culture of medium spiny neurons and midbrain DA neurons using a microfluidic device, recapitulating the nigrostriatal pathways which are disrupted in PD. This represents an interesting model for future studies, especially as the central channel, where the axons and synapses extend, is capable of hosting a separate environment to that of the lateral channels housing the cell soma. However, the problem of cell heterogeneity and probable contamination of neuronal cultures with subtypes of different lineages again raises the need for single-cell transcriptomics in order to accurately assess the populations being produced by these differentiation protocols. Excitingly, tri-culture systems encompassing human iPSC-derived microglia, astrocyte and neurons have now been described (Guttikonda et al., 2021). This tri-culture system has been utilised to examine cellular cross-talk in cells harbouring the APP*^swe+/+^* mutation in order to elucidate neuroinflammatory mechanisms in Alzheimer’s disease, however it is only a matter of time before similar systems are generated from PD patient-derived iPSC.

**TABLE 1:** Factors used in the derivation of midbrain cultures from human embryonic stem cells (ESC) and induced pluripotent stem cells (iPSC).

Factors	Role in midbrain development	References
SB431542	Used for TGFβ/activin/nodal and BMP pathways inhibition, and also brachyury (mesoderm) i.e., dual SMAD inhibition, to differentiate neuroectoderm.	Chambers et al., 2009
LDN193189	Used in conjunction with SB431542 for dual SMAD inhibition. LDN193189 inhibits BMP and also SOX17 (endoderm). Homologs of LDN193189 such as dorsomorphin and Noggin are also commonly used	Chambers et al., 2009
SHH-C24II	Specify midbrain floor plate identity through ventralisation/floor-plate induction	Nolbrant et al., 2017
CHIR99021	Specify midbrain floor plate identity, activates WNT signalling, GSK3 inhibitor, important for caudalisation, promotes proliferation of midbrain neural progenitor cells	Kriks et al., 2011; Sarrafha et al., 2021
FGF8b	Expressed at midbrain/hindbrain boundary, specifies midbrain floor plate identity through precise caudalisation	Nolbrant et al., 2017
Purmorphamine	SHH agonist, ventral floor-plate specification	Nolbrant et al., 2017
Smoothened Agonist	SHH pathway agonist, ventralisation/floor-plate induction	Denham et al., 2012

iPSC represent exciting opportunities for PD research, both at a mechanistic as well as clinical level. The ability to use these cells for drug screening opens up many avenues for PD therapeutics and advancement of clinical trials. The ability to test drugs on PD patient-derived cells *in vitro*, with different genetic mutations as well as idiopathic PD may help to identify subpopulations that are resistant to certain drugs and can help tailor the treatment process for patients, helping to get the most effective drug to the patient as quickly as possible.

#### Potential Limitations of Induced Pluripotent Stem Cells as a Parkinson’s Disease Model System

As outlined above with human ESC, a limitation of iPSC is the problem of replicating aged populations. During reprogramming, iPSC are reverted back to a pluripotent state and thus lose important epigenetic and age-associated changes (Maherali et al., 2007; Horvath, 2013; Mertens et al., 2015; Kerepesi et al., 2021). Currently age is inferred onto the cells using long-term culture or artificial induction, interestingly Kerepesi and colleagues have recently demonstrated that passaging of pluripotent cells does not increase their epigenetic age from zero (Kerepesi et al., 2021). However, the ability to bypass the pluripotent state in reprogramming of adult human fibroblasts toward a neuronal lineage has been demonstrated (Ladewig et al., 2012; Liu et al., 2013) and would prevent the loss of these epigenetic and age-associated characteristics of PD patient-derived cells. Although, these cells do have a limited “shelf-life” and become senescent with long-term culture.

Line-to-line variability is another challenge when working with iPSC, this becomes a particular problem when it comes to transplantation. There is poor characterisation of heterogeneous populations produced, there is need to standardise differentiation protocols, however this is difficult due to the ever changing and developing field where advancements are made regularly to provide a more accurate differentiation of cells from specific brain regions. There is an increased recognition of the importance of in-depth analysis and consistency of differentiated populations and thus a large uptake in the use of technology such as RNA-sequencing for single-cell transcriptomics to tackle this problem. This is important as it has been demonstrated that heterogeneous cell populations will respond differently to the same genetic mutations (Fernandes et al., 2020).

However, this does bring up the fact that iPSC-derived cell populations do not fully recapitulate the intercellular interactions of *in vivo* populations. They may solve the issue of genetics whereby disease specific gene doses and mutant proteins are more representative of the human condition compared to rodent models, however a lack of yet unidentified CNS factors in these cultures maybe limit the ability to generate appropriate phenotypes.

The defined populations generated from differentiation of iPSC allow for thorough analysis of specific cellular responses to insult and injury however intercellular relationships cannot be examined in this manner, and the culture requirements for the various neural cell populations leave co-culture systems difficult to maintain. iPSC-derived organoids offer a means to model complex human tissues while also recapitulating features of architecture, composition and function. Organoids may help overcome some of the limitations associated with single cell populations in iPSC-derived culture models.

## Brain Organoid Models of Parkinson’s Disease

The advent of organoids is considered a major breakthrough in stem cell research and has enabled advancements in the applications of human iPSC for disease modelling. Human heart organoids provide an *in vitro* model of cardiac development and congenital heart disease (Lewis-Israeli et al., 2021), inner ear organoids have been generated for modelling deafness (Romano et al., 2021), airway epithelial cell organoids are frequently used in lung research (Eenjes et al., 2021), intestinal organoids enable *in vitro* investigations of gut pathology (Puschhof et al., 2021), and it is even possible to derive sensorimotor organoids capable of forming neuromuscular junctions (Pereira et al., 2021).

The generation of organoids is based on the ability of iPSC to self-organise, just as ESC do in the developing embryo. The first protocol for the generation of brain organoids was published in 2013 and described the methods to produce cerebral organoids to model development and microcephaly (Lancaster et al., 2013). This initial protocol avoided the use of extrinsic patterning and growth factors in order to avoid limiting development of brain regions. These non-directed brain organoids were self-organising with intrinsic signalling, therefore produced cells from numerous brain regions with spontaneous cell commitment. These regions included cells with forebrain, midbrain and hindbrain identities, as well as retinal tissue identities. They even developed fluid filled spaces comparable to brain ventricles (Lancaster et al., 2013). However non-directed brain organoids generate highly variable regionalised populations, and if a specific region is desired this can mean few cells are produced with the appropriate regionality. Protocols now exist for the guided differentiation of midbrain specific organoids which are more suited for modelling disorders of the midbrain, including PD. This guided differentiation strategy is also proposed to reduce line-to-line, and batch-to-batch variability (Nickels et al., 2020). Some of these protocols and the major findings from midbrain organoid research are summarised in Table 2.

**TABLE 2:** Summary table of the protocols for the generation of midbrain organoid models and the main findings from these model systems.

Model and methods	Main findings	References
Midbrain organoids supported by spider-silk microfibers functionalised with laminin	Silk organoids reproduce key molecular aspects of DA neurogenesis and reduce intra-organoid variability in DA formation and cell type composition.	Fiorenzano et al., 2021
Midbrain organoids generated by automated processes	Protocol for the automated production of neural progenitor cells (NPC) in midbrain organoids. System controls seeding, splitting and expansion of human fibroblasts, iPSC and NPC to produce sufficient cell numbers to carry out high throughput screening.	Boussaad et al., 2021
Human PSC-derived midbrain dopaminergic neurons	Protocol for the generation of 3D midbrain organoids with a high level of homogeneity using PSC containing a TH-TdTomato reporter. At differentiation D30, 30% of the organoid cell population were TH-TdTomato^+^.	Sarrafha et al., 2021
Human NESC-derived midbrain organoids	Protocol for the derivation of midbrain organoids from neuroepithelial stem cells (NESC) using an embedding technique for reproducibility. Organoids were shown to contain functional neurons and glial cells, and can be maintained in long-term culture.	Zagare et al., 2021
Human ESC-derived midbrain organoids with *DNAJC6* mutations	Mutant *DNAJC6* was introduced into ESC using CRISPR-Cas9 gene editing. These mutant lines were used to produce midbrain organoids. Mutant *DNAJC6* resulted in DA neuron degeneration, α-syn aggregation and mitochondrial and lysosomal dysfunction in midbrain-like organoids.	Wulansari et al., 2021
Midbrain organoids with mutant Parkin	Midbrain organoids derived from patient iPSC carrying *PRKN* mutation had a reduced overall size and decreased numbers of astrocytes compared to control, and fragmentation of TH^+^ neuronal processes, an indication of degeneration.	Kano et al., 2020
Human midbrain organoids derived from ESC	*In vivo*-like midbrain organoids. Mesencephalon identity was most robust with the combination of dorsomorphin, A83-01 and CHIR99021. Organoids structurally and functionally mature; 86% TH^+^ and they produced dopamine. DA neurons susceptible to MPTP toxin via astrocyte mediated conversion to MPP^+^.	Kwak et al., 2020
Human midbrain organoids	Optimised approach for the standardised generation of midbrain organoids with DA neurons and astrocyte differentiation without core cell death and suitable for whole-mount imaging. 6-OHDA significantly reduces the number of DA neurons in a dose dependent manner, but does not affect astrocytes.	Nickels et al., 2020
PBMC-derived iPSC used to generate midbrain organoids	The first midbrain organoid model of idiopathic PD. Reduced TH^+^ neurons, potentially as a result of an early reduction in *FOXA2* and *LMX1A* expression. Increased expression of *PTX3*.	Chlebanowska et al., 2020
PSC-derived midbrain spheroids carrying the *POLG1 p.Q811R* variant	Oligomeric α-syn was found in *POLG1 p.Q811R* spheroids. Mass-spectrometry based quantitative proteomic analysis identified altered pro- and anti-inflammatory signalling. Glycolysis was increased in *POLG1 p.Q811R* spheroids.	Chumarina et al., 2019
Isogenic midbrain organoids ± *LRRK2 G2019S* mutation	TXNIP is required for the development of LRRK2-associated PD pathology in midbrain organoids.	Kim et al., 2019
PD patient-derived midbrain organoids carrying *LRRK2 G2019S* mutation	Reduced number and complexity in DA neurons in PD organoids compared to control. Increased FOXA2 in PD organoids compared to control; might *LRRK2 G2019S* mutation affect DA neuron development?	Smits et al., 2019
Human midbrain organoids derived from human NESC	Midbrain organoids show neuronal, astroglial, and oligodendrocyte differentiation, contain spatially organized groups of dopaminergic neurons, synaptic connections, electrophysiological activity, and myelination. 64% of neurons express DA markers TH^+^/FOXA2^+^/LMX1A^+^ confirming midbrain identity.	Monzel et al., 2017
Human PSC-derived midbrain-like organoids	Large multicellular organoid-like structure that contains distinct layers of neuronal cells expressing characteristic markers of human midbrain. They secrete DA, produce neuromelanin, form functional synapses and exhibit neuron-like electrophysiological properties. At D60 22% of MAP2^+^ neurons were also TH^+^.	Jo et al., 2016
Human midbrain-like organoids generated using γ-secretase inhibition and varying culture times	Protocol for the size calibrated, directed differentiation of midbrain organoids. Controlled maturation and cellular composition of 3D engineered nervous tissue; rapid production (3 weeks) of >60% of cells with midbrain-like phenotype (FOXA2, LMX1A, TH, NURR1, and EN1).	Tieng et al., 2014

Brain organoids generally consist of neural stem cells (NSC), neurons and astrocytes. After prolonged development oligodendrocyte progenitor cells and oligodendrocytes may develop within the organoid. This is representative of primitive brain development in the embryo whereby a population of NSC develop from ectoderm which give rise to neurons first followed by astrocyte progenitor cells and oligodendrocyte progenitor cells which will mature into astrocytes and oligodendrocytes respectively. Microglia follow a different developmental pathway, as with embryonic development they are generated from the mesoderm germ layer and so will not develop in brain organoids. However, they may be separately differentiated from iPSC and later introduced to produce a tri-culture system with neurons and astrocytes capable of mimicking the interactions between these cell types (Lin et al., 2018; Guttikonda et al., 2021).

Brain organoids of a variety of brain regions have been generated to date, including the cortex (Lancaster et al., 2013; Lancaster and Knoblich, 2014) and cerebellum (Muguruma et al., 2015), while midbrain-like organoids can also be produced (Tieng et al., 2014; Monzel et al., 2017; Zagare et al., 2021).

### Generation of Midbrain Organoids

Early protocols utilised the self-organising nature of pluripotent stem cells (PSC) to generate brain organoids, but as mentioned previously this results in the development of numerous regionalised populations. The generation of midbrain-specific organoids requires the addition of patterning factors in order to promote the development of specialised structures resembling the midbrain. These specialised organoids are generated from an already regionally patterned population of NSC with ventral neural tube fate. Similar to the derivation of midbrain populations in 2D cultures, this requires the timely addition of specific factors involved in midbrain development, namely SMAD inhibitors, CHIR99021 and SHH (Table 1).

The process of midbrain organoid development typically begins with the generation of embryoid bodies. This is achieved by transitioning PSC from their classical adherent 2D cultures into a suspension culture, thereby allowing the PSC to form a bundle of cells similar to the early embryo–an embryoid body. Dual-SMAD inhibition induces neuroectoderm differentiation and enriches the embryoid body with primitive neural tissue by inhibiting differentiation of mesoderm and endoderm tissues. SB431542 is used to inhibit the Activin/TGFβ signalling pathway. Noggin, or its homologs dorsomorphin and LDN193189, are used to inhibit the BMP signalling pathway. The combination of SB431542 and either of the BMP antagonists achieves dual-SMAD inhibition and in turn complete neural conversion (Chambers et al., 2009). To control the regional specification of the neural progenitor cells (NPC) in the organoids the application of additional patterning factors is required to ensure a midbrain fate. Expansion of neuroepithelium within embryoid bodies is achieved with the addition of CHIR99021 and purmorphamine which stimulate the WNT and SHH signalling pathways respectively (Reinhardt et al., 2013). In the developing midbrain, activation of the WNT pathway results in the expression of the transcription factor *LMX1A* (an early midbrain marker) (Yang et al., 2013). SHH, which is secreted by the floor plate, and subsequent activation of the SHH pathway leads to expression of *FOXA2* (Bayly et al., 2012). *LMX1A* and *FOXA2* in turn act as developmental factors and induce the expression of a plethora of genes required for later development of the midbrain (Chung et al., 2009). To date, numerous approaches for the generation of midbrain organoids have been published most of which apply the traditional embryoid body to organoid approach, however some protocols now use bioprinting and bioreactor-based techniques. Protocols for the generation of human midbrain organoids are summarised and their key findings highlighted in Table 2.

### Midbrain Organoids Offer an Attractive Alternative to Traditional 2D Culture Systems

Brain organoid models offer an attractive alternative to 2D culture models; an innovative 3D culture system which recapitulate some of the complex characteristics and physiology of the human midbrain that are lacking in 2D models. The combination of 3D culture techniques (e.g., Aggrewells and ultra-low adherence culture flasks) and human iPSC facilitate the production of 3D culture models. They may provide the missing link between *in vitro* 2D models and *in vivo* animal models. Brain organoids have already been successfully used to model development (Lancaster et al., 2013), Alzheimer’s disease (Lin et al., 2018; Ghatak et al., 2019), and Creutzfeldt-Jakob disease (Groveman et al., 2019). The ability to maintain organoids for long-term culture, up to 1 year, means that they may be used for aging studies, and/or long-term drug interventions.

Brain organoids could be used as a more relevant model in drug screening studies and potentially help reduce the current clinical trial failure rate by providing a physiologically relevant pre-clinical model for high throughput screening (Boussaad et al., 2021). Due to the multifactorial nature of neurodegenerative diseases such as PD the use of single cell type models might limit target discovery and the potential for phenotypic screening using animal models can also be limited due to species differences (Burns et al., 2015). Brain organoids provide a human based 3D culture system that may combat the limitations of other models in the drug screening process. In addition, since iPSC carry the same genetic signature as the patient they are derived from, then the generated organoids will express any pathogenic mutations present.

#### Midbrain Organoids Model Anatomical and Physiological Features of the Midbrain

Midbrain organoids contain several features which effectively model the anatomy and physiology of the human midbrain. These include the presence of multiple cell populations in a 3D architecture as well as physiologically relevant functional characteristics such as dopamine production and release.

Midbrain organoids express markers associated with the human midbrain including FOXA2, NURR1, PITX3, and TH (Qian et al., 2016). Midbrain organoids contain different subtypes of neuronal cells and complicated architecture of the midbrain. Kwak et al. generated organoids with a FOXA2^+^ ventricular zone, an ASCL1^+^ and LMX1A^+^ intermediate zone, and TH, MAP2, and DAT triple positive marginal zone which replicates the structures of the developing midbrain. These organoids also contained mature midbrain DA neurons (Kwak et al., 2020). Midbrain organoids contain multiple brain cell types therefore relate to the human midbrain environment. They have been shown to contain up to 60% DA neurons and 20% astrocytes (GFAP^+^ S100β^+^ cells) (Zagare et al., 2021). In the developing embryo, NPC and neurons develop prior to glial cells. Midbrain organoids have been shown to follow this developmental trajectory with the emergence of astrocytes occurring around day 60 of differentiation (Monzel et al., 2017). The presence of myelin sheath-forming oligodendrocytes has also been observed (Monzel et al., 2017). Kwak and colleagues have also reported the presence of multiple cell types within their organoids. They found that DA neurons were co-localised with GABAergic neurons as is the case in the midbrain. The presence of glutamatergic neurons, as well as astrocytes and oligodendrocytes was also confirmed (Kwak et al., 2020). The presence of multiple cell types mean that brain organoids facilitate the analysis of cell-to-cell interactions which may be involved in the pathogenesis of PD. Furthermore, neurons in 3D cultures have a greater level of connectivity than is capable in 2D culture systems and therefore the level of complexity of the neuronal networks is more closely related to that of the adult human brain (Smits and Schwamborn, 2020), therefore organoid cultures provide an anatomically suitable model of the midbrain.

Human brain organoids mimic actual brain biology, thereby lending themselves as an ever more popular research model. Physiological features of the midbrain include electrophysiologically functional neuronal networks, dopamine production and release, presence of other neuronal subtypes including glutamatergic and GABAergic neurons. Functional analyses can be carried out on these physiologically active organoids. Within midbrain organoids, neurons have been shown to develop myelination and synaptic connections, furthermore they show normal firing patterns and network synchronicity (Smits and Schwamborn, 2020). Monzel et al. (2017) provided evidence of dopamine production and spontaneous electrical activity in their midbrain organoids. Brain organoids with the characteristics of normal brain function provide a physiologically accurate representation of this brain region which might help further elucidate the mechanisms at play in PD. Kwak and colleagues have also provided evidence of dopamine production in their midbrain organoids which peaked at week 20. They also demonstrated electrical activity in their organoids and furthermore showed that the cells were capable of producing neuromelanin (Kwak et al., 2020). A key feature of a subpopulation of DA neurons is the expression of neuromelanin, and its presence is typically associated with maturation of these cells (Zecca et al., 2001). Sarrafha et al. (2021) confirmed the presence of neuromelanin in DA neurons in long-term cultured organoids (D200 +) using Fontana-Masson and tyrosine hydroxylase staining. Neuromelanin is a pigment expressed by neurons in the SN and locus coeruleus and is characteristically lost in PD (Haining and Achat-Mendes, 2017). Neuromelanin is not typically found in 2D cultures of PSC-derived DA neurons, however this protein has been found to be produced by the cells in a number of midbrain organoid protocols (Jo et al., 2016; Monzel et al., 2017). The presence of neuromelanin provides another advantage for the use of organoid cultures as they have features which more closely represent the true physiology of these cells (Galet et al., 2020).

#### Patient-Derived Organoids Are Capable of Replicating Features of Parkinson’s Disease Pathology

Midbrain organoids have been shown to replicate many features of PD pathology, including degeneration of DA neurons, α-syn production, phosphorylation and accumulation, ROS production and oxidative stress, and they have even been shown to mimic pathology as a result of PD-associated mutations [for review see Smits and Schwamborn (2020)]. Therefore, they hold great promise for modelling the aetiology of PD. To this end, a small number of studies have generated organoids from human iPSC that carry a mutation associated with PD, namely *PINK1*, *PARKIN* and *LRRK2-G2019S*, to study the impact of background genetics on organoid development, functionality and cell interactions. One such study, Brown et al. (2021) demonstrated that global neuronal differentiation did not differ between PINK1 deficient organoids and their isogenic controls but dopamine neurogenesis was impeded in the PINK1 deficient organoids. In addition, two studies have examined organoids carrying the *LRRK2-G2019S* mutation. Smits et al. (2019) using both patient-derived or genetically modified midbrain organoids carrying the *LRRK2-G2019S* mutation, demonstrated PD relevant phenotypes which included a reduced number of midbrain DA neurons and a significant reduction in the complexity of these DA neurons. In addition, Kim et al. using organoids with the same mutation in *LRRK2*, demonstrated reduced expression of TH, aromatic L-amino acid decarboxylase (AADC) and DA transporter (DAT) and an increase in caspase-3 compared to relevant isogenic control. Furthermore, this *LRRK2-G2019S* mutation resulted in an increase in phosphorylated α-syn in the area of Thioflavin positive deposits. Interestingly, when mutant midbrain organoids were treated with the LRRK2 kinase inhibitor GSK2578215A, this reduced the accumulation of phosphorylated α-syn, decreased dopamine neuronal cell death and so the expression levels of TH, AADC and DAT were partially restored (Kim et al., 2019). These studies highlight the potential for the use of patient specific organoids to both increase our understanding of the occurrence and progression of PD pathology and also highlights their usefulness in drug discovery.

Human ESC with a loss of function mutation have also been used to generate midbrain organoids. The loss-of-function mutations in the *DNAJC6* gene, which encodes HSP40 auxilin, has been linked with early-onset forms of PD (Olgiati et al., 2016) and midbrain organoids with this mutation showed DA neuron degeneration, α-syn aggregation and mitochondrial and lysosomal dysfunction. Furthermore, *DNAJC6* mutation organoids had compromised development due to impairment of the WNT-LMX1A pathway during the early development of the organoids. This led the authors to theorise that *DNAJC6* mutations leave midbrain DA neurons vulnerable to degeneration in PD as a result of improper development (Wulansari et al., 2021). Interestingly, Smits and Schwamborn (2020) reported that midbrain organoids carrying the *LRRK2-G2019S* mutation, had an increase in the floor plate marker FOXA2. Given that FOXA2 is required for midbrain DA neuron development, the authors suggested that there could be a neurodevelopmental defect in midbrain DA neurons expressing *LRRK2-G2019S*. Taken together both these studies could be outlining a neurodevelopmental defect associated with these mutations but further studies would be required to elucidate this.

As mitochondrial dysfunction is a very prominent area of research in PD pathogenesis it is crucial to have an understanding of how such processes can be investigated in organoid models. A recent study by Duong et al. (2021) demonstrated the generation of iPSC and subsequently cerebral organoids from human PBMC and found that this process did not alter the genomic integrity of the mitochondria from that of the donor cells. Furthermore, they were able to assess the functionality of the mitochondria within the organoids and demonstrate their capability to respond to pharmacological interventions. This is important as it would allow generation of patient specific organoids to study disease-associated effects on mitochondrial function within PD.

The cells within midbrain organoids are susceptible to neurotoxin much like their *in vivo* counterparts. MPTP is converted to MPP^+^ in the CNS by astrocytes and subsequently causes degeneration of midbrain DA neurons thereby acting as a neurotoxin for induction of PD symptoms in animal models. Due to the lack of astrocytes and neuron-glial interactions in many *in vitro* cultures MPP^+^ is typically used in these cases to induce DA neuron death. In order to test the efficacy of cell-glial interactions in midbrain organoids, Kwak et al. (2020) administered MPTP and observed significant death of TH^+^ cells in their organoids. This indicates that the astrocytes within the organoids are functional and thereby facilitate the neurotoxic action of MPTP. Patient-derived iPSC expressing mutant Parkin (encoded by *PRKN*) were used to generate midbrain organoids (Kano et al., 2020) and to study the astrocytes therein. Results demonstrated decreased numbers of GFAP positive astrocytes and an increase in TH fragmentation in *PRKN*-mutated organoids compared to aged and sex matched controls which could suggest a non-cell autonomous role of astrocytes in DA neuron cell death (Kano et al., 2020). The ability of midbrain-like organoids to replicate aspects of PD pathology *in vitro* has huge translational applications and could provide a model for drug screening. Furthermore, the patient specific nature of organoids could also open up avenues for patient specific therapies.

#### Potential Limitations of Midbrain Organoid Models

The generation of iPSC, their expansion and the long-term maintenance of derived cultures and organoids is an extremely expensive process in terms of cost as well as being time intensive (Kuo et al., 2020). A number of inhibitors, patterning factors and growth factors are required for cell maintenance and contribute to the cell culture bill. In addition, the developmental processes mimicked by the differentiation protocols often require long-term maintenance of these cultures to produce mature cell populations which further contributes to the cost of iPSC-based models.

PSC cultures and their derivatives have high batch-to-batch variability. The reproducibility of iPSC production and their subsequent differentiation has been a longstanding limitation of the field. Factors that impact reproducibility include the transfection and differentiation protocols used, as well as the experience of the people performing the protocols (Volpato and Webber, 2020). A study published in Stem Cell Reports in 2018 uncovered variability between laboratories when tasked with the differentiation of the same two iPSC lines following the same protocol. The potential causes of such variation was attributed to passage number and the storage conditions of progenitors for the differentiation of neurons (Volpato et al., 2018). Emerging strategies are aimed at circumventing batch-to-batch variability and include automated systems for the expansion and differentiation of iPSC-derived models (Kane et al., 2019; Pandey et al., 2019; Boussaad et al., 2021).

PSC-derived midbrain organoids often have a low efficiency, and result in immature and heterogeneous structures which might impact translational ability since an uneven dispersal of DA neurons throughout the organoids is not representative of the midbrain. Furthermore, the lack of vascularisation in organoids leads to oxygen and nutrient deprivation of the cells in the centre of the organoid; the core. This can result in necrosis which has been observed in many protocols for organoid generation—not just brain organoids (Lancaster et al., 2013; Ramachandran et al., 2015; Monzel et al., 2017). Bioengineered scaffolds to support cell organisation (Lancaster et al., 2017), bioreactors (Qian et al., 2016), as well as microfluidic devices (Cho et al., 2021) and use of an air-liquid interface culture (Giandomenico et al., 2019) promote survival and maturation of brain organoids and reduce variability. Problems with nutrient supply and oxygenation can be circumvented by culturing organoids in millifluidic cultures achieved by a spinning bioreactor or on orbital shaker plates, both of which allow organoids to grow up to 4 mm in diameter (Lancaster and Knoblich, 2014; Berger et al., 2018). By optimising neuronal induction, preparing organoids from a committed cell type at a reduced seeding density and with an accelerated differentiation protocol Nickels et al. (2020) prevented the development of a necrotic core. Efforts are also being made to generate vascularised brain organoids (Mansour et al., 2018; Cakir et al., 2019; Shi et al., 2020). Protocols which produce organoids within a few months might generate cells with an immature phenotype and therefore lack the age signature which will in turn impact PD relevant pathology. Improved techniques for the generation and maintenance of organoids such as those mentioned above will hopefully allow for long-term culture of organoids in order to generate a more relevant “aged” phenotype in the cell populations.

Midbrain organoids do not contain all the cell types of the human midbrain. In general, it has been noted that there is a paucity of glial cells in midbrain organoids (Jo et al., 2016; Qian et al., 2016), and quite notably microglia are often missing from these 3D cultures owing to a different developmental pathway to neurons, astrocytes and oligodendrocytes. Some efforts have been made to separately differentiate microglia and later incorporate these cells into brain organoids (Song et al., 2019b). Similarly, efforts have been made to generate brain organoids that include endothelial cells and pericytes (Cakir et al., 2019; Song et al., 2019a) and as mentioned above vascularised brain organoids have been produced so it is simply a matter of time before these protocols are translated to midbrain organoids. Additionally, the advent of vascularised midbrain organoids may facilitate the modelling of blood-brain barrier breakdown and infiltration of peripheral immune cells, both of which are key pathological features of PD (Brochard et al., 2009; Gray and Woulfe, 2015; Sommer et al., 2018; Al-Bachari et al., 2020; Pediaditakis et al., 2021) and important features of a complex *in vitro* model.

## Contributions of Human Brain-Based Models to the Field

**FIGURE 1:**
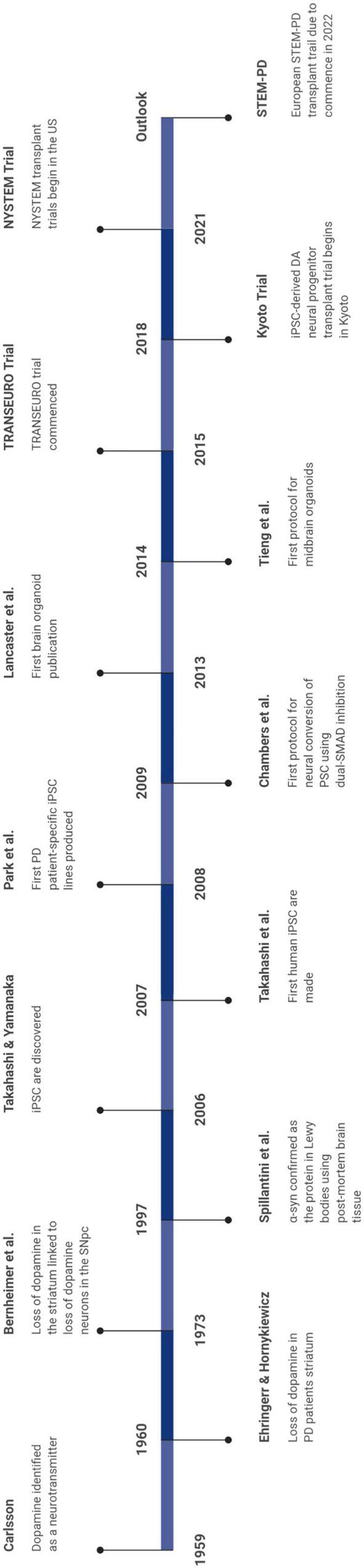
Timeline denoting the major advancements in the field.

Human brain-based models of PD have contributed greatly to our understanding of disease mechanisms and pathogenesis, while also facilitating a means to test viable drug candidates (Bolognin et al., 2019; Klima et al., 2021; Zanetti et al., 2021). Some of the major breakthroughs in the PD field as a result of research using human brain-based models are noted in Figure 1.

iPSC-derived DA neurons were utilised in a transcriptomic study which found that a number of subsets of DA neurons exist (Fernandes et al., 2020). Single cell RNA-seq analysis identified two progenitor populations and four categories of DA neurons, denoted DAn1-4, which have distinct genetic profiles. It was found that DAn1 neurons are more susceptible to rotenone-induced oxidative stress. From this, it was found that cholesterol and synaptic genes could be used as potential indicators of stress response. Furthermore, it was found that DAn1 neurons were also more susceptible to tunicamycin-induced ER-stress, in turn increasing expression of genes associated with mRNA processing, DNA repair, histone modification, and methylation. The application of human brain-based models for in depth investigations of the cell populations affected in PD will hopefully help identify new targets for therapeutic intervention.

Human PSC not only facilitate production of human brain-based test systems but have also got therapeutic potential. The first cell transplant trials for people with PD began in the 1980s and 1990s (Barker et al., 2013). Early protocols used foetal ventral mesencephalic tissue, and more recently foetal tissue is being utilised in the TRANSEURO trial which commenced in 2015 (NCT01898390) (Barker, 2019). A number of ongoing clinical trials are underway with human ESC-derived neural precursor cells, as well as DA neurons, being used as the graft tissue. There are currently ongoing clinical trials aiming to investigate the safety and efficacy of human ESC-derived neural precursor cells for the treatment of PD (NCT03119636, NCT02452723) (Garitaonandia et al., 2016; Wang et al., 2018). The clinical approaches being applied are injection of the cells directly into the striatum, or the combined injection of cells into the striatum and SN. Trials using cells derived from human iPSC are also underway. The Kyoto trial (UMIN000033564) which began in 2018 utilises HLA-matching; they have generated human iPSC-derived DA NPC from a healthy control patient with the most common HLA type in Japan, with the aim that this will reduce the immune reaction to the grafts once injected into patients based on encouraging pre-clinical data (Doi et al., 2020; Takahashi, 2020). The idea of HLA-matching is to reduce the potential immune complications between recipient and donor graft (Morizane et al., 2017), however immunosuppressants are still used in this trial as they are not individually HLA-matched (Takahashi, 2020). Ideally, an autologous transplant approach would be the best option for minimising any immune-reaction to the graft, and in 2020 Schweitzer and colleagues demonstrated in a single patient case study that autologous transplant of human iPSC-derived DA NPC into the putamen was achieved without the need for immunosuppressants thereafter (Schweitzer et al., 2020). In 2021, the NYSTEM trial began in the US utilising human ESC-derived midbrain DA neurons to target the putamen [NCT04802733 (Studer, 2017; Gravitz, 2021)] following success of preclinical efficacy and safety characterisation of their human ESC (Piao et al., 2021). And on the European front, 2022 will see the start of the STEM-PD trial (Kirkeby et al., 2017). Overall, there is lots of exciting work underway, to elucidate the efficacy of human brain-based therapeutics, that leaves us more hopeful than ever, that cell transplantation may be a viable option for Parkinson’s in the future.

## Conclusion

Undoubtably seminal studies with post-mortem tissue paved the way for many more, opening up a whole new field of research using *in vitro* cell lines, animal models and more recently stem cell models; all of which have increased our understanding of the mechanisms of cell death in PD and in so doing continue to lead to the development of neuroprotective and neurorestorative therapies. Midbrain organoids provide an environment in which PD pathology can develop and be examined, therefore provide an extremely powerful model system for the study of PD. The continued use of human brain-based models will hopefully provide the insight required to produce more and better therapies for PD patients.

## Author Contributions

All authors listed have made a substantial, direct, and intellectual contribution to the work, and approved it for publication.

## Conflict of Interest

The authors declare that the research was conducted in the absence of any commercial or financial relationships that could be construed as a potential conflict of interest.

## Publisher’s Note

All claims expressed in this article are solely those of the authors and do not necessarily represent those of their affiliated organizations, or those of the publisher, the editors and the reviewers. Any product that may be evaluated in this article, or claim that may be made by its manufacturer, is not guaranteed or endorsed by the publisher.
